# Coagulation in gout: is there a link with disease activity?

**DOI:** 10.1007/s10067-022-06047-9

**Published:** 2022-02-01

**Authors:** Daisy Vedder, Martijn Gerritsen, Joost C. M. Meijers, Michael T. Nurmohamed

**Affiliations:** 1 Amsterdam Rheumatology & Immunology Center, Reade, Amsterdam, Netherlands; 2 Amsterdam Cardiovascular Sciences, Vrije Universiteit, Amsterdam, Netherlands; 3 Department of Experimental Vascular Medicine, University of Amsterdam, Amsterdam UMC, Amsterdam, Netherlands; 4 Department of Molecular Hematology, Sanquin Research, Amsterdam, Netherlands; 5 Department of Rheumatology, Amsterdam UMC, Amsterdam, the Netherlands

**Keywords:** Cardiovascular disease, Coagulation, Disease activity, Gout, Inflammation

## Abstract

**Objective:**

To investigate the coagulation system in gout patients and associations between disease activity and levels of coagulation markers.

**Methods:**

A prospective cohort study was performed with data from 30 Dutch gout patients. Levels of coagulation markers including APTT, PT, D-dimer, prothrombin F1 + 2, von Willebrand factor, and thrombin generation parameters were analyzed at baseline and 1-year visit. These markers were related to clinical markers of gout disease activity including the Gout Activity Score (GAS). Our hypothesis was that patients with gout and active disease have increased levels of coagulation markers and that a decrease in disease activity would lead to normalization of coagulation activity.

**Results:**

A higher GAS was associated with increased levels of thrombin generation parameters including ETP (*ß* = 0.48, *p* = 0.01), peak thrombin (*ß* = 0.60, *p* = 0.001), and velocity index (*ß* = 0.57, *p* = 0.002). Tophaceous gout and higher SUA levels were associated with thrombin generation parameters. After 1 year, thrombin generation parameters showed a small procoagulant trend despite a moderate decrease in disease activity. Prospectively measured changes in disease activity according to the GAS were not associated with any of the coagulation markers.

**Conclusion:**

Patients with active gout have higher levels of thrombin generation markers, indicating a link between disease activity and coagulation. A change in disease activity after 1 year was not associated with significant changes in coagulation markers, probably due to prolonged low-grade inflammation. Future studies should focus on levels of coagulation markers in comparison with the general population and the effect of adequate gout treatment.

**Table:** 

Key Points *• Patients with gout have an increased risk of cardiovascular events.* *• High disease activity was associated with higher levels of thrombin generation markers.* *• Over time, small decreases in inflammation were associated with a decrease in D-dimer and thrombin generation.*

## Introduction

Gout is the most common inflammatory arthritis with a prevalence of 1–4% in Europe and its incidence is still rising [1, 2]. If not treated adequately, gout evolves into a more chronic and disabling disease. Gout is often accompanied by comorbidities, predominantly cardiovascular disease. A large retrospective cohort study in the UK showed a significantly increased risk of any arterial vascular event in males, with an absolute risk of 44 events per 1000 patient years in comparison with 34 events in male patients without gout (*HR* 1.22, 95% *CI* 1.16–1.29). This was even higher in female gout patients (52 vs 33 events per 1000 person years, *HR* 1.45, 95% *CI* 1.34–1.57) [3]. These events include angina pectoris, myocardial infarction, cerebrovascular accidents, and peripheral vascular disease. Two large cohort studies in Taiwan also showed an increased risk of 40–70% (5.3 vs 2.6 events per 1000 person years) to develop venous thromboembolism in patients with gout (*HR* 1.38, 95% *CI* 1.18–1.62 and *HR* 1.66, 95% *CI* 1.37–2.01, respectively) [4, 5]. Surprisingly, the relative risk was the highest among patients in the youngest age group (20–49 years) (*HR* 2.04, 95% *CI* 1.24–3.37). This increased risk is largely independent of traditional risk factors, and therefore, a causative link between gout and cardiovascular events has been suggested. To date, the underlying pathophysiologic mechanisms have not been fully elucidated. It is clear that inflammation in inflammatory rheumatic disorders leads to endothelial dysfunction, which itself has been linked to activation of the coagulation system [6]. In other inflammatory rheumatic diseases such as rheumatoid arthritis (RA), chronic inflammation increases the risk of thromboembolic processes by upregulating procoagulants, downregulating anticoagulants, and suppressing fibrinolysis [7, 8].

Coagulation studies in gout patients are scarce. Two retrospective studies investigated levels of fibrinogen and D-dimer in patients with gout as possible markers of gout disease activity. Both studies showed increased levels of these markers in comparison with a matched control group. Fibrinogen and D-dimer were associated with gout disease activity, defined as the number of flares and Gout Activity Score (GAS), suggesting a link between gout and coagulation activation [9, 10]. However, to date, no prospective coagulation gout studies have been published.

Therefore, the aim of our study was to investigate, prospectively, the coagulation system in gout patients and its relationship with disease activity. Our hypothesis was that (1) patients with active gout have a more procoagulant state and (2) that a decrease in disease activity would lead to normalization of coagulation activity.

## Materials and methods

### Patients’ characteristics

A pilot study was performed with data from the first 30 gout patients included in the “Reade gout cohort Amsterdam.” This cohort includes patients referred to the rheumatologist in the previous 3 months before entering the study who fulfilled the 2015 gout classification criteria according to the ACR and EULAR collaborative initiative [11]. Patients were included between 2015 and 2019. There were no exclusion criteria. Rheumatologists were asked to approach newly diagnosed gout patients for participation in the gout cohort. All referred patients were included in the cohort.

At baseline and after 1 year, data regarding demographics, disease duration, disease activity, comorbidities, and medication use were collected. Disease activity was assessed by the number of flares in the last year, number of involved joints (tender and swollen), presence of tophi, serum uric acid level, and inflammation markers (CRP and ESR). The definition of a flare required the presence of ≥ 3 out of 4 criteria (patient-defined gout flare, pain at rest > 30 on a 0–100 numeric rating scale, presence of at least one swollen joint, presence of at least one warm joint, as validated in earlier studies) [12]. Patients’ perspective on disease activity was assessed using the Visual Analog Scale (VAS0-10) for pain and disease activity. Four variables were used to calculate the Gout Activity Score (GAS), including the number of flares in the last 12 months, SUA, number of tophi, and VAS disease activity (GAS3-step-c) [13]. Physical examination included vital parameters, tender and swollen joint count, and laboratory variables.

The study was approved by the ethics committee board (METC Slotervaart and Reade Amsterdam). Written informed consent was obtained from all participants. Patients and the public were not involved in the design of this study.

### Laboratory tests

Sampling was performed during baseline and 1-year visit, and citrate plasma samples were immediately centrifuged for 15 min at 3000 rpm (1860 g). Plasma was stored at − 80 °C until further use. Measurement of coagulation parameters was performed at the Department of Experimental Vascular Medicine, Academic Medical Center, University of Amsterdam, after completion of the study. A set of coagulation markers was chosen based on earlier findings in other rheumatic diseases including markers representing endothelial activation (von Willebrand factor) and coagulation (APTT, PT, prothrombin fragment 1 + 2, D-dimer) [8]. Moreover, we added thrombin generation assays (TGA) because of their additive contribution in understanding and evaluating the overall hemostatic processes [14].

All measurements were performed in a single batch. von Willebrand factor (vWF) antigen was measured by a homemade ELISA (antibodies from DakoCytomation, Denmark) (reference range: 50–150%). Prothrombin fragment 1 + 2 was determined by ELISA from Siemens Healthcare Diagnostics (53–271 pMol/L). Prothrombin time (PT) (10.7–12.9 s), activated partial thromboplastin time (aPTT) (25.0–38.0 s), and D-dimer (< 1.00 mg/L) were measured by BCS-XP (Siemens Healthcare Diagnostics) using reagents and methods of the manufacturer. In vitro thrombin generation initiated by 1 pM tissue factor was determined by calibrated automated thrombinography (CAT) (Thrombinoscope BV) [15]. The following parameters were derived from the thrombograms: lag time, peak thrombin, time-to-peak, velocity index, and endogenous thrombin potential (ETP). Peak thrombin, velocity index, and ETP were normalized to normal pooled plasma obtained from more than 250 healthy individuals.

### Statistics

Data were analyzed using SPSS Statistics 27. Patients’ characteristics were expressed as number (percentage), means (± standard deviation (SD)), when normally distributed, and otherwise median (interquartile range IQR), when skewed distributed. Distribution was explored with the use of visualization techniques (histograms and Q-Q plots) and when not conclusive, analyzed with the Shapiro–Wilk test (cut off *p* < 0.05). Linear regression analysis was used for associations between gout disease activity and levels of coagulation markers. Log transformation was performed for skewed data, and when unsuccessful, the Mann–Whitney *U* test was used instead of linear regression analysis. Because of the performance of multiple tests (with 10 different markers of coagulation), we applied a Bonferroni correction for all analyses performed at baseline. Therefore, the threshold of significance was set at *p* = 0.005 (0.05/10 = 0.005).

After crude analysis, all results were adjusted for age and gender when needed. A paired sample *t*-test was performed to compare disease activity and levels of coagulation markers at baseline with those at the 1-year visit. Furthermore, we looked into possible associations for differences in disease activity between two visits and increase/decrease in coagulation markers.

Patients with a history of coagulation disorders or the use of anticoagulants during the study were excluded from all analyses involved in that particular coagulation pathway.

## Results

### Patient characteristics

The patients were almost all male with a mean age of 54 years. Gout characteristics are shown in Table 1. All patients experienced an acute flare in 6 weeks before inclusion. Approximately, one-quarter of the patients still experienced painful and/or swollen joints during the baseline visit. Mean serum uric acid was 0.44 mmol/L (7.33 mg/dL). Nineteen patients (60%) used colchicine during the last gout flare (dose 0.5–1.5 mg/day). Two-thirds of the patients used NSAIDS, while half of the patients were treated with corticosteroids during an attack in the last year. More than half of the patients (*N* = 17, 57%) were using urate-lowering therapy (allopurinol *N* = 17) at the baseline visit. After 1 year, all patients had a follow-up visit. Gout disease activity according to the GAS, number of flares in the last 12 months, VAS pain, and VAS disease activity were significantly lowered at the follow-up visit (resp. *p* = 0.004 and *p* < 0.001). Nine patients used NSAIDS and nine patients reported the use of colchicine between baseline and 1-year visit from whom three patients used colchicine (0.5 mg) temporarily as flare prevention during initiation of urate-lowering therapy. Sixteen patients (53%) were using urate-lowering therapy (allopurinol *N* = 14, benzbromarone *N* = 2) at the time of the 1-year visit. Three patients started urate-lowering therapy (ULT) after the baseline visit. Four initial users stopped their ULT treatment between the two visits. Mean SUA level after 1 year was 0.42 mmol/L (7.00 mg/dL).

**Table 1:** Patient characteristics of the gout cohort

Characteristics of the gout cohort	Baseline visit (*N* = 30)	1-year visit (*N* = 30)	*p*-value difference
Sex (*N*, % male)	29 (97)	29 (97)	-
Age in years (mean, SD)	54 (12)	55 (12)	-
Disease duration in years (median, IQR)	3 (2–8)	4 (3–9)	-
Crystal proven gout (*N*, % yes)	13 (43)	14 (47)	-
Gout Activity Score (mean, SD)	4.2 (1.1)	3.2 (0.8)	*p* < 0.001
Amount of gout flares last year (median, IQR)	3 (2–5)	2 (0–3)	*p* < 0.001
Subcutaneous tophaceous gout (*N*, %)	9 (30)	9 (30)	*p* = 1.00
VAS pain (median, IQR)	20 (4–49)	0 (0)	*p* = 0.004
VAS disease activity (median, IQR)	23.5 (4–49)	0 (0)	*p* < 0.001
Patients with painful joints (*N*, %)	8 (27)	3 (10)	*p* = 0.18
Patients with swollen joints (*N*, %)	7 (23)	1 (3)	*p* = 0.07
Serum urate (mmol/L) (mean, SD) (mg/dL) (mean, SD)	0.44 (0.08) 7.33 (1.33)	0.42 (0.10) 7.00 (1.67)	*p* = 0.23
CRP (mg/L) (median, IQR)	2 (1–4)	2 (2–5)	*p* = 0.38
ESR (mm/H) (median, IQR)	7 (2–12)	6 (2–10)	*p* = 0.87
Systolic blood pressure (mmHg) (mean, SD)	137 (13.2)	142 (17.8)	*p* = 0.22
Smoking, yes (*N*, %)	6 (20)	4 (13)	
BMI (mean, SD)	29.6 (4.0)	29.6 (4.1)	*p* = 0.99
Cholesterol ratio (total cholesterol/HDL-cholesterol) (mean, SD)	4.5 (1.6)	4.2 (1.1)	*p* = 0.19

### Coagulation markers

Samples from twenty-seven patients were used for the analysis of coagulation markers. Three samples were excluded from all analyses except vWF due to the presence of hemophilia (*N* = 1) or the use of anticoagulants (*N* = 2). Table 2 shows the characteristics of the coagulation markers. There were small differences between baseline and the 1-year visit in mean/median values of almost all coagulation markers.

**Table 2:** Characteristics of coagulation markers

Coagulation marker	Gout cohort baseline	1-year visit	Reference values	*p*-value difference
APTT in s (mean, SD)	31.9 (4.3)	31.1 (3.4)	25.0–38.0	*p* < 0.01
PT in s (mean, SD)	11.5 (0.5)	11.4 (0.6)	10.7–12.9	*p* < 0.01
D-dimer mg/L FEU (median, IQR)	0.28 (0.18–0.58)	0.29 (0.2–0.41)	< 1.00	*p* = 0.14
vWF in % (mean, SD)	122.9 (44.0)	131.7 (59.6)	50–150	*p* = 0.29
F1 + 2 in pMol/L (mean, SD)	197.7 (85.1)	180 (59.5)	53–271	*p* = 0.17
*Thrombin generation assays*				
Lag time in min (median, IQR)	4.7 (4.0–5.3)	4.5 (4.1–5.0)		*p* = 0.19
Peak height thrombin in % (mean, SD)	81.4 (19.6)	89.2 (22.3)		*p* < 0.01
Time to peak in min (mean, SD)	10.0 (1.8)	9.4 (1.7)		*p* = 0.05
ETP in % (mean, SD)	93.7 (12.7)	97.8 (14.9)		*p* = 0.04
Velocity index in % (mean, SD)	73.7 (35.4)	85.9 (38.4)		*p* = 0.01

### Coagulation and disease activity at baseline visit

#### Clinical markers of disease activity

**Fig. 1:**
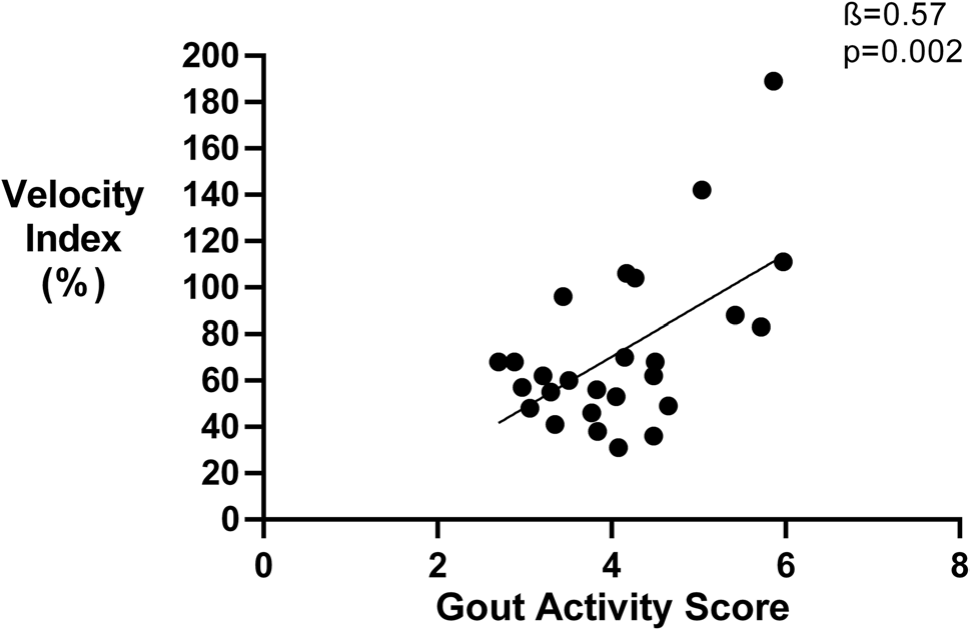
Disease activity according the Gout Activity Score is associated with Velocity index

**Fig. 2:**
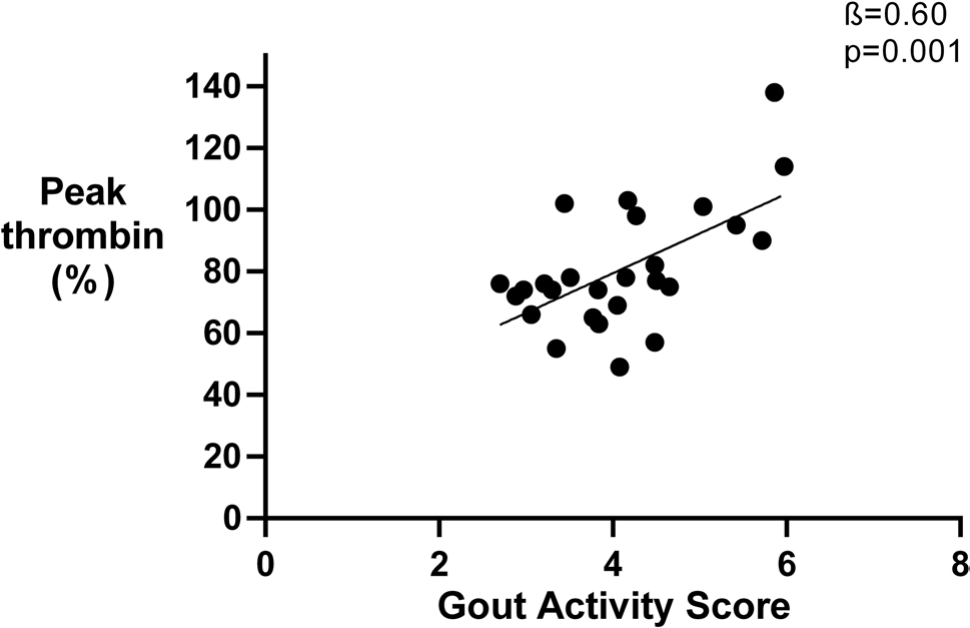
Disease activity according the Gout Activity Score is associated with Peak thrombin levels

Different markers of clinical disease activity during baseline visit were analyzed in relation to markers of coagulation activation. First of all, a higher GAS at baseline was associated with higher levels of three thrombin generation markers, including ETP (*ß* = 0.48, *p* = 0.01), velocity index (*ß* = 0.57, *p* = 0.002, Fig. 1), and peak thrombin (*ß* = 0.60, *p* = 0.001, Fig. 2). The associations with velocity index and peak thrombin remained significant after correction for multiple testing. Separately, VAS disease activity level was associated with a higher velocity index (*ß* = 0.51, *p* = 0.006) and peak thrombin (*ß* = 0.55, *p* = 0.003). Tophaceous gout was also associated with a higher velocity index (*ß* = 0.39, *p* = 0.05), whereas higher levels of SUA were related with a higher ETP (*ß* = 0.374, *p* = 0.05) and peak thrombin (*ß* = 0.38, *p* = 0.05). The last component of the GAS, the number of flares in the last year, was not associated with any of the coagulation markers. After correction for multiple testing associations between VAS disease activity and thrombin generation, markers remained significant.

Other disease activity variables such as the number of painful or swollen joints during the baseline visit were associated with higher levels of ETP (*ß* = 0.38, *p* = 0.05) but not significant after correction for multiple testing. The current use of urate-lowering therapy (yes/no) or the use of colchicine during the last attack was not associated with any of the coagulation markers.

#### Inflammatory markers

Higher levels of ESR were associated with an increase in lag time (*ß* = 0.46, *p* = 0.02) and D-dimer (*ß* = 0.58, *p* = 0.002). The association with D-dimer remained significant after correction for multiple testing. Other coagulation markers were not significantly associated with the level of inflammation.

### Comparison baseline and 1-year visit

When we compared the baseline coagulation values with the 1-year visit values, a statistically significant decrease was seen in aPTT and PT (resp. *p* = 0.009 and *p* = 0.005). An increase was observed in three thrombin generation assays, although differences were small (ETP (*p* = 0.04), velocity index (*p* = 0.01), and peak height (*p* = 0.007)), and not significant after correction for multiple testing. All other coagulation markers were not significantly different after 1 year.

As stated, disease activity according to the GAS was slightly lower at the 1-year visit compared to baseline (− 0.97 points, *p* < 0.001). A decrease in the GAS was not associated with a significant decrease in the thrombin generation markers or any other coagulation markers.

**Fig. 3:**
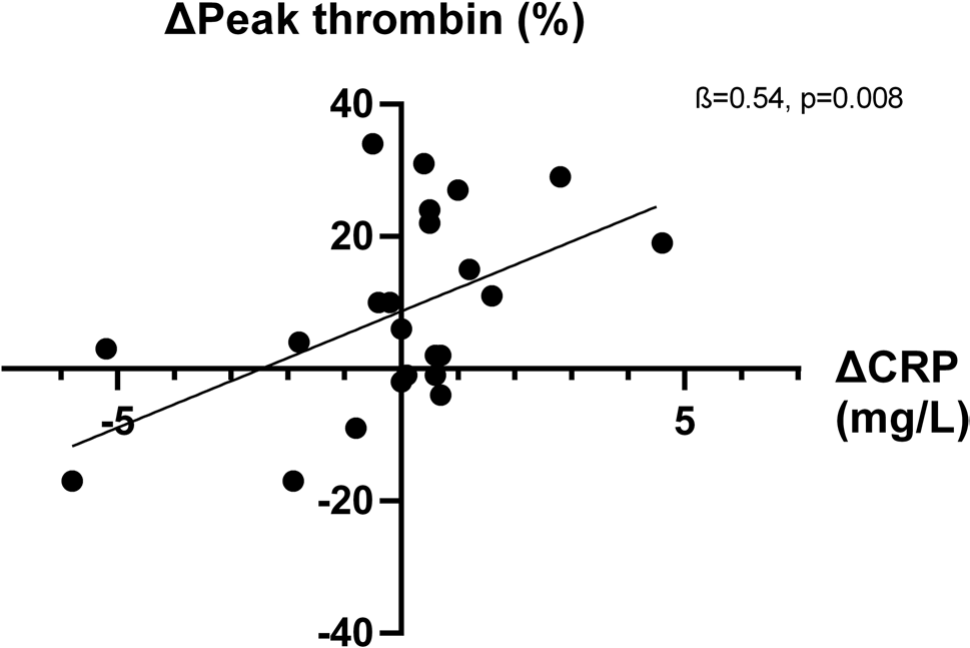
A decrease  in CRP was associated with a decrease in Peak thrombin

**Fig. 4:**
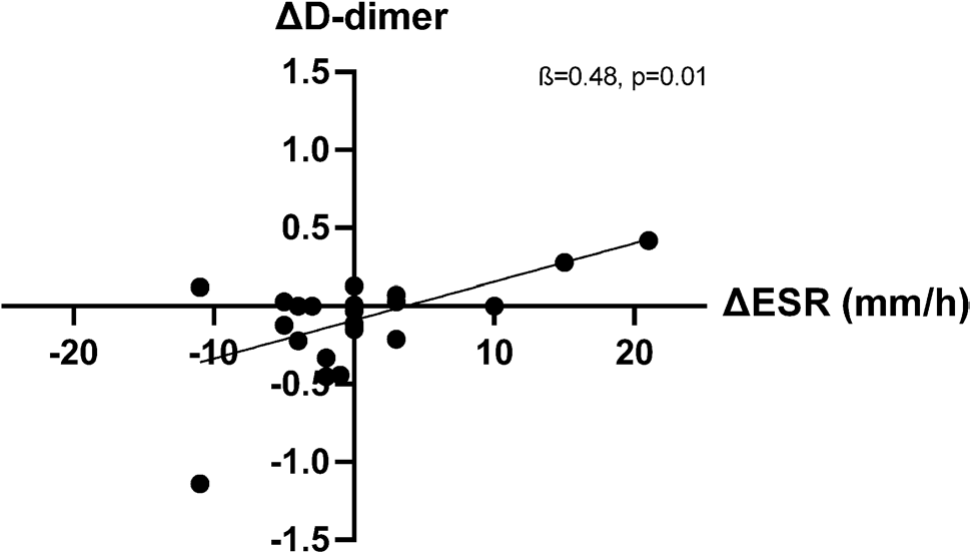
A decrease in ESR was associated with a decrease in D-dimer

Overall differences in inflammatory markers were small except for one patient (∆*CRP* =  + 37, ∆*ESR* =  + 21). After excluding this outlier, we looked into an association between differences in inflammation (∆CRP and ∆ESR) and differences in coagulation between the visits. A positive association was seen between ∆CRP and ∆velocity index (*ß* = 0.48, *p* = 0.02) as well as ∆peak thrombin (*ß* = 0.53, *p* = 0.008, Fig. 3). Furthermore, a decrease in ESR was associated with a decrease in D-dimer (*ß* = 0.48, *p* = 0.01) (Fig. 4).

## Discussion

Our study showed a number of interesting associations at baseline suggesting a link between gout and coagulation. These associations were almost all with the GAS (or GAS components) and markers of thrombin generation. Earlier studies show that high levels of thrombin are associated with more dense and stable fibrin networks and these denser fibrin clots are thought to increase the risk of thrombosis [16]. Thrombin generation markers give a better representation of the physiological state in comparison with more routine assays such as PT/APTT and this could be the reason that we did not find an association with these markers.

Our second objective was to see if a decrease in disease activity after 1 year was associated with a decrease in coagulation activation. First, a lower disease activity was confirmed by a (small) decrease in GAS (− 0.97), a significant decrease in the number of painful and swollen joints, and significantly lower scores on VAS pain and disease activity. Second, coagulation markers overall did not change after 1 year, except for an increase in thrombin generation markers, which actually reflects a more procoagulant state after 1 year.

Differences in disease activity between the visits were not associated with differences in coagulation markers, which could be due to a limited decrease in gout disease activity. Inflammatory markers were comparable at both visits and serum uric acid levels remained high (> 0.36 mmol/L, > 6 mg/dL). One hypothesis is that patients in our cohort have prolonged chronic low-grade inflammation due to insufficient gout treatment that translated into persistent low-grade coagulation activation. A limitation of this study is the fact that inflammatory markers were only determined at two time points (baseline and 1 year) and therefore do not reflect inflammatory state during the year. Although elevated inflammatory markers at both time points could suggest a chronic inflammatory state, future studies with samples at more time points in between should rule out incidental finding in this pilot study.

With the exception of thrombin generation markers, we did not find strong associations with other coagulation markers such as von Willebrand factor and prothrombin F1 + 2. A possible explanation for this negative finding could be insufficient levels of inflammation at baseline. Although most patients experienced a gout flare in the weeks before their first visit, inflammatory markers were relatively low at baseline . In other rheumatic diseases, inflammation was linked with endothelial activation, and in future studies, it would be interesting to analyze samples which are taken at the beginning of and during the flare.

Besides disease activity, another interesting question is whether  the use of flare treatment, particularly colchicine, was associated with (de)activation of the coagulation system. Colchicine has anti-inflammatory properties and could therefore possibly lower coagulation activity. The use of colchicine in our cohort was not associated with levels of coagulation markers. Most patients used colchicine only for the duration of a flare with a cumulative daily dose between 0.5 and 2.0 mg.

The strengths of this study are the well-defined cohort, diversity of patient data collected, and the extent of coagulation markers which were studied at two time points. The most important limitation of this study is the conscious choice for a small exploratory pilot study without the addition of a control group. Therefore, the results were only compared within patients between the two time points and not compared with the general population. Although mean/median levels of the different coagulation markers fell within reference values, it is possible that a direct comparison with male patients in the same age category would show differences that would have strengthened the evidence for activation of certain coagulation pathways. A second limitation is the rather small population, although this was one of the first prospective studies investigating coagulation in gout patients. The next step would be to perform a cohort study with a matched control group and to focus on the process of coagulation initiation, particularly thrombin generation. Another interesting follow-up study would be to focus on coagulation at the commencement and during a gout flare and to compare patients with adequate ULT treatment with inadequate or no treatment.

## Data Availability

Data are available upon reasonable request.
