# Correction to: Coagulation in gout: is there a link with disease activity?

**DOI:** 10.1007/s10067-022-06102-5

**Published:** 2022-03-04

**Authors:** Daisy Vedder, Martijn Gerritsen, Joost C. M. Meijers, Michael T. Nurmohamed

**Affiliations:** 1 Amsterdam Rheumatology & Immunology Center, Reade, Amsterdam, Netherlands; 2 Amsterdam Cardiovascular Sciences, Vrije Universiteit, Amsterdam, Netherlands; 3 Department of Experimental Vascular Medicine, University of Amsterdam, Amsterdam UMC, Amsterdam, Netherlands; 4 Department of Molecular Hematology, Sanquin Research, Amsterdam, Netherlands; 5 Department of Rheumatology, Amsterdam UMC, Amsterdam, the Netherlands

**Correction to: Clinical Rheumatology**

**https://doi.org/10.1007/s10067-022-06047-9**

In the original version of the above article, The figures and legends used were incorrect. The correct figures are presented as follows (Figs.1, 2, 3, and 4):

**Fig. 1:**
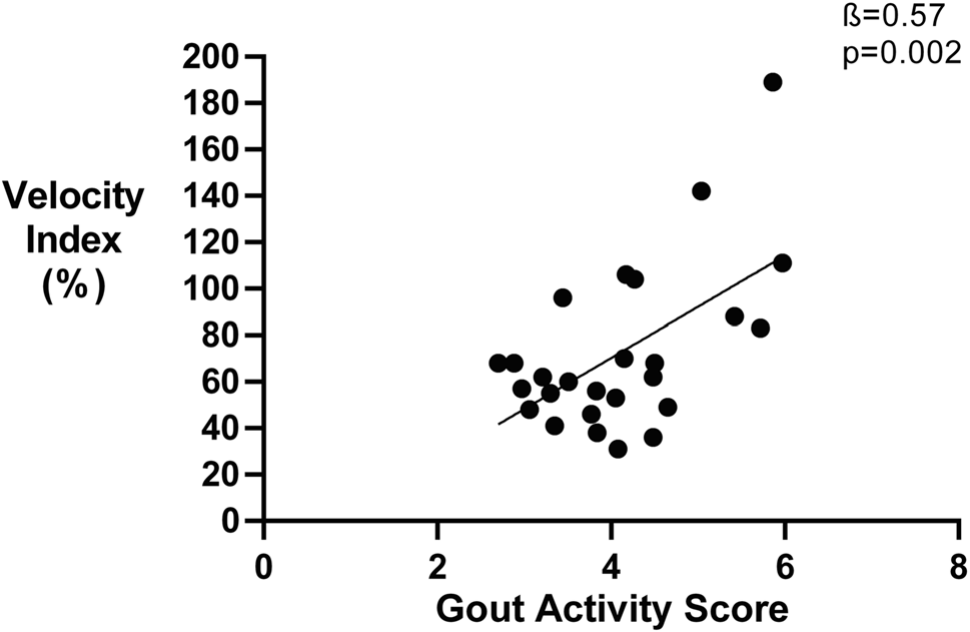
Disease activity according the Gout Activity Score is associated with Velocity index

**Fig. 2:**
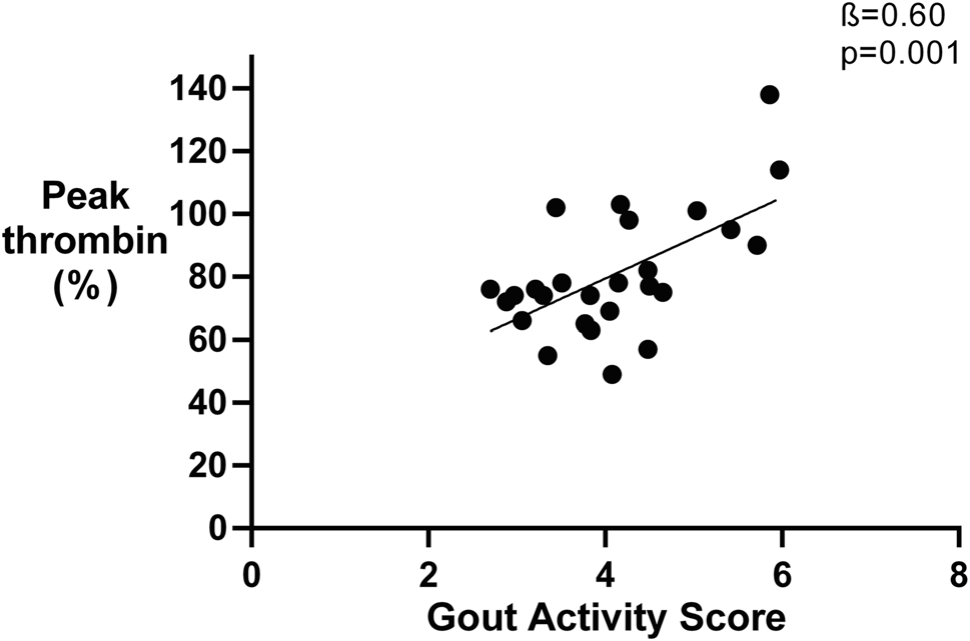
Disease activity according the Gout Activity Score is associated with Peak thrombin levels

**Fig. 3:**
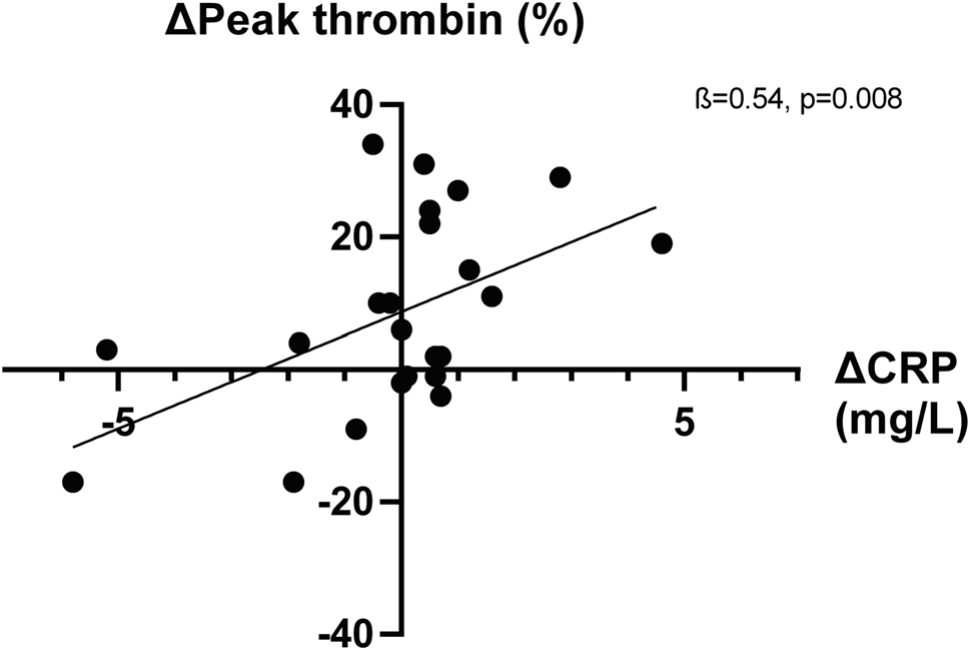
A decrease in CRP was associated with a decrease in Peak thrombin

**Fig. 4:**
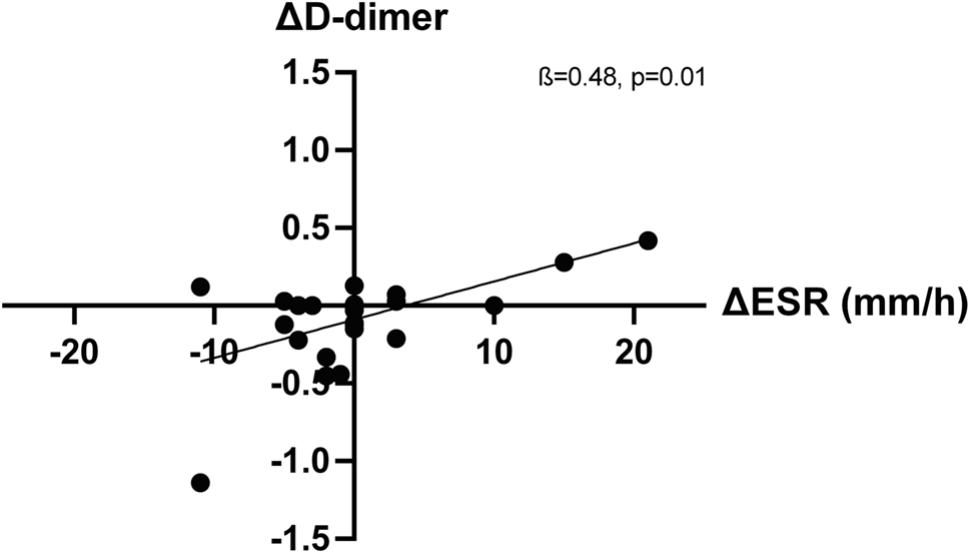
A decrease in ESR was associated with a decrease in D-dimer

The original article has been corrected.

